# Quantitative Determining of Ultra-Trace Aluminum Ion in Environmental Samples by Liquid Phase Microextraction Assisted Anodic Stripping Voltammetry

**DOI:** 10.3390/s18051503

**Published:** 2018-05-10

**Authors:** Liuyang Zhang, Jinju Luo, Xinyu Shen, Chunya Li, Xian Wang, Bei Nie, Huaifang Fang

**Affiliations:** 1 Key Laboratory of Analytical Chemistry of the State Ethnic Affairs Commission, College of Chemistry and Materials Science, South-Central University for Nationalities, Wuhan 430074, China; zlyzly1239@163.com (L.Z.); JinjuLuo@163.com (J.L.); lichychem@163.com (C.L.); xwang27@hotmail.com (X.W.); bnie@cigit.ac.cn (B.N.); 2 Key Laboratory of Analytical Chemistry for Biology and Medicine, Wuhan University, Ministry of Education, Wuhan 430072, China; shenxy@whu.edu.cn

**Keywords:** ionic liquid, liquid phase microextraction, anodic stripping voltammetry, aluminum ion

## Abstract

Direct detecting of trace amount Al(III) in aqueous solution by stripping voltammetry is often frustrated by its irreversible reduction, resided at −1.75 V (vs. Ag/AgCl reference), which is in a proximal potential of proton reduction. Here, we described an electroanalytical approach, combined with liquid phase microextraction (LPME) using ionic liquid (IL), to quantitatively assess trace amount aluminum in environmental samples. The Al(III) was caged by 8-hydroxyquinoline, forming a superb hydrophobic metal–chelate, which sequentially transfers and concentrates in the bottom layer of IL-phase during LPME. The preconcentrated Al(III) was further analyzed by a square-wave anodic stripping voltammetry (SW-ASV). The resulting Al-deposited electrodes were characterized by scanning electron microscopy and powder X-ray diffraction, showing the intriguing amorphous nanostructures. The method developed provides a linear calibration ranging from 0.1 to 1.2 ng L^−1^ with a correlation coefficient of 0.9978. The LOD attains as low as 1 pmol L^−1^, which reaches the lowest report for Al(III) detection using electroanalytical techniques. The applicable methodology was implemented for monitoring Al(III) in commercial distilled water.

## 1. Introduction

Spectrometric techniques, such as atomic absorption spectrometry [1], inductively coupled plasma atomic emission spectrometry [2], fluorometry [3], and inductively coupled plasma–mass spectrometry (ICP-MS) [4], are widely applied in detecting metals in aqueous samples. Alternatively, electroanalytical techniques offer important advantages, such as good sensitivity, high selectivity, cost-effective, easy for automation and suitable for portable devices [5,6]. However, in aqueous solutions, direct voltammetric determination of metal ions with high electronegativity, such as aluminum, potassium, sodium and barium, is limited due to the parallel hydrogen evolution reaction. 

Polarographic determination of Al(III) was achieved by reducing aluminum di-o-hydroxyazo complex [7]. Codeposition with Zn^2+^ on screen-printed carbon electrode prior to differential-pulse anodic stripping voltammetry (ASV) was also adopted [8]. However, the sensitivities of these methods remain poor. The voltammetric response of an electroactive ligand that forms a complex with Al(III) were changed in the presence of Al(III), which provides an indirect electrochemical strategy for the determination of Al(III) [9,10,11,12,13,14,15,16,17,18,19,20,21,22,23,24,25,26,27,28,29,30,31,32,33,34,35,36,37,38,39,40,41]. There also are a few biosensors developed for Al(III) [42,43,44]. Among these reports, the lowest detection limit was 8 pmol L^−1^ of Al(III), using electrochemical impedance spectroscopy [45].

Many ionic liquids have the following unique physical properties: wide electrochemical windows, high thermal and chemical stability, negligible vapor pressure, good extractabilities for various organic compounds and metal ions, and high conductivities compared to non-aqueous solvents. Because of these characteristics, ILs are employed in multiple fields such as batteries [46], fuel cells [47], electrochemistry [48], catalysis [49], synthesis [50] and electroplating [51]. In addition, several methods, combining extraction using ionic liquid with suitable analytical methods, have been developed to determine trace and sub-trace levels of metal ions [52,53,54,55,56]. Due to the high viscosity of ILs, diluting with organic solvents or back-extraction should be taken prior to analysis [52]. For back-extraction, several acidic and basic solutions have been used as stripping medium. This additional step is usually time-consuming, and also a potential source of contamination, which limits its wide utilization. 

IL-based liquid–liquid extraction (LLE) combined with electrochemistry was first reported by Hussey group: the Cs^+^ and Sr^2+^ in aqueous samples were selective extracted into ionic liquid and was analyzed at a mercury film electrode [57]. The IL-based LLE coupled with ASV to determine Pb(II) and Cd(II) was also reported by Nagaosa [58]. The extraction of Pb using IL followed by differential pulse ASV on a boron-doped diamond microcell was presented by Jaffrezic-Renault et al. [59]. The calibration curves for Pb are in the range of 0–4 μg L^−1^, with a LOD of 0.3 μg L^−1^. Recently, Mercury was extracted with IL using temperature controlled dispersive liquid phase microextraction (LPME) and then detected by ASV [60]. Mercury was enriched by 17 times and a LOD of 0.05 μg L^−1^ was acquired. However, there is to date no report that IL-based LLE coupled with ASV to determine the metal ions with high electronegativity.

In the present work, an LPME–SW-ASV method has been developed for the determination of ultra-trace Al(III) in aqueous samples. In our experiments, the extraction agent was 1-octy-3-methylimidazolium hexafluorophosphate [C_8_mim][PF_6_], and the chelating agent was oxine. The factors influencing LPME efficiency and SW-ASV signals were systematically studied. The method developed can determine Al(III) in environmental samples with upgraded sensitivity and better selectivity, which also can be applied to determine other active metal ions.

## 2. Materials and Methods

### 2.1. Reagents and Solutions

All chemicals were of analytical reagent grade unless stated otherwise. All solutions used were prepared using ultrapure water obtained by a Milli-Q Advantage A10 system (Millipore, Bedford, MA, USA). The experiments were carried out at room temperature (approximately 25 °C). Stock standard solution of Al(III) were prepared by dissolving appropriate amounts of aluminum nitrate (purity ≥ 99.99%, Aladdin Reagent Co., Shanghai, China) in hydrochloric acid. The samples were obtained by diluting the stock standard sample with proper solvents. 1-octy-3-methylimidazolium hexafluorophosphate [C_8_mim][PF_6_] (purity ≥ 99%) (Chengjie Chemical Reagent Co., Shanghai, China) was employed as an extracting agent without further purification. 8-hydroxyquinoline was purchased from Merck (Darmstadt, Germany). The stock solution of 10 mmol L^−1^ oxine was prepared by dissolving the appropriate amount of oxine in ethanol and was kept in refrigerator (4 °C) for a week. Metal solutions (CuSO_4_·5H_2_O, MgCl_2_·6H_2_O, ZnSO_4_, Cr(NO_3_)_3_·9H_2_O, CaCl_2_, Cd(NO_3_)_2_ and SnCl_2_ (Sinopharm Chemical Reagent Co. Ltd., Shanghai, China) were used for interference experiments; HNO_3_, CH_3_COOH (Sinopharm Chemical Reagent Co. Ltd., Shanghai, China) were used as received; sodium acetate (purity ≥ 99.99%) was obtained from Aladdin (Aladdin Reagent Co., Shanghai, China). 

### 2.2. Instrumentation 

All electrochemical measurements were carried out using a CHI-660D electrochemical station (Chenhua Instruments Co., Shanghai, China). The peak current after a blank subtraction was used to perform linear regression. Data was processed with Origin 7.0 software. Scanning electron microscopy (SEM) images were obtained by a quanta 200 scanning electron microscopy equipped with energy dispersive spectroscopy (FEI, Hillsboro, OR, USA). The X-ray diffractograms were recorded using a Bruker D8 Advance using monochromatized Cu Ka radiation. A TGL-20B centrifuge with a 30° fixed angle rotor (rmin = 6.5 cm, rmax = 11 cm) (Shanghai Anting Instrument Factory, Shanghai, China) was adopted to perform centrifugation. The pH of solution was measured with MS-H-S meter (Dragon Instrumentation Co. Ltd., Beijing, China). The three electrode system, consisting of a gold disc working electrode (O.D. 3 mm), a Pt auxiliary electrode, and an Ag/AgCl (solid) reference electrode was used. Voltammetric measurements were carried out in a microliter voltammetric cell (MVC) (Figure S1) (Supplementary Materials) in a Faraday cage. The MVC was made from polytrifluorochloroethylene. The diameter of the bottom hole was 1.2 cm, and the diameter of the top hole (from 4 mm to 9 mm) can be adjusted according to the volume of the extractant during LPME (from 10 µL to 400 µL). As shown in Figure S1 (Supplementary Materials), the working electrode was set at the bottom of the MVC, the Pt electrode was screwed into MVC horizontally, and the Ag/AgCl solid electrode was put into the MVC vertically. The vertical distance between Au electrode and Pt electrode was 0.5 mm. All potentials were given with respect to the Ag/AgCl solid electrode. To avoid accidental contamination, all sample vials and containers used were soaked in (1 + 1) nitric acid over 24 h, and rinsed with copious amounts of ultrapure water.

### 2.3. LPME Procedures

The 30 mL water samples, containing 0.246 g of sodium acetate, were placed into 50.0 mL conical-bottom polypropylene vials, and then a given amount of oxine was added as chelating agent. After mixing, the sample was placed in the dark for 20 min to complete the complexation. Then, a 150 µL volume of [C_8_mim][PF_6_] was added into each sample vial. The Al (III)-oxine complex was extracted from water samples into IL phase during centrifugation (at 4500 rpm for 10 min). After extraction, the IL phase was collected, and 100 µL of extractant was transferred into the MVC for the subsequent SW-ASV analysis.

### 2.4. Measurement Procedure

The gold disk electrode was polished to a mirror-like smoothness using 0.05 µm Al_2_O_3_ powder. It was washed successively with 1:1 (*v*/*v*) HNO_3_, ultrapure water, ethanol and ultrapure water in an ultra-sonic bath and then dried in air. The procedures for the determination of Al(III) by SW-ASV were as follows: 100 µL of extractant was transferred into the MVC. Accumulation was achieved at a given deposition potential for a fixed time. The voltammogram was recorded by applying a positive-going square-wave voltammetric potential scan from −1.6 V to 0 V (with a frequency of 20.0 Hz, pulse amplitude of 25.0 mV, and a step potential of 4.0 mV). After the measurement, a 60 s clean step at potential of 0.3 V was used to remove possible residual metals. No deaeration of solutions was needed in our SW-ASV experiments. 

### 2.5. Determination of Al(III) in Real Samples

The concentration of Al(III) in Wahaha^®^ (Hangzhou, China) pure distilled water was determined by LPME–SW-ASV. Then, 2.46 g of sodium acetate was dissolved in 300 mL of Wahaha^®^ pure distilled water and the pH of solution was adjusted to 6.5 with acetic acid. A 0.3 mL of sample solution was introduced into 2 mL conical-bottom polypropylene vials, then 20 µL oxine (10 mmol L^−1^ in ethanol) was added as chelating agent. A 150 µL volume of [C_8_mim][PF_6_] was added in each sample vial. In order to eliminate matrix effects, all commercial distilled water samples were analyzed using standard addition method. For evaluation measurements, an ICP-MS unit model NexIon300X (PerkinElmer, Richmond, CA, USA) was used.

## 3. Results and Discussion

### 3.1. Electrodeposition and Characterization of the Aluminum Deposits on Gold Disk Electrode

**Figure 1:**
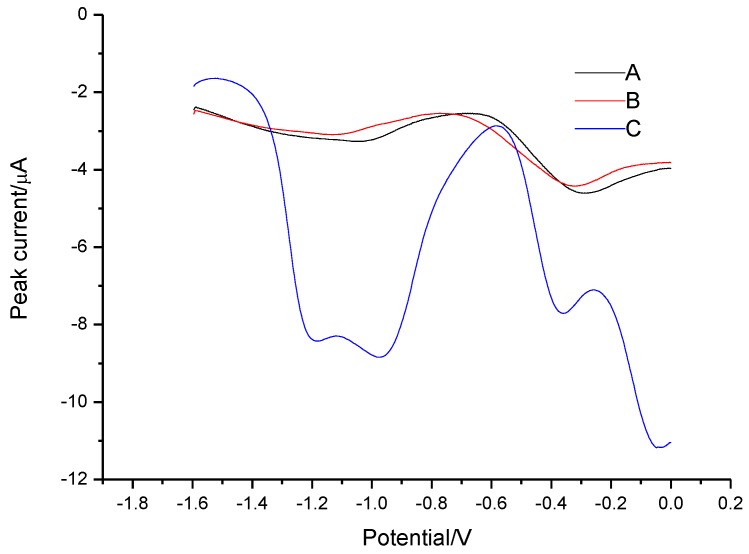
The square-wave anodic stripping voltammetry (SW-ASV) on gold disk electrode (GDE) at different condition: (A) [C_8_mim][PF_6_]; (B) oxine in [C_8_mim][PF_6_]; (C) IL-based liquid phase microextraction (LPME) of 0.4 ng L^−1^Al(III) in presence of 0.67 mmol L^−1^ oxine.

**Figure 2:**
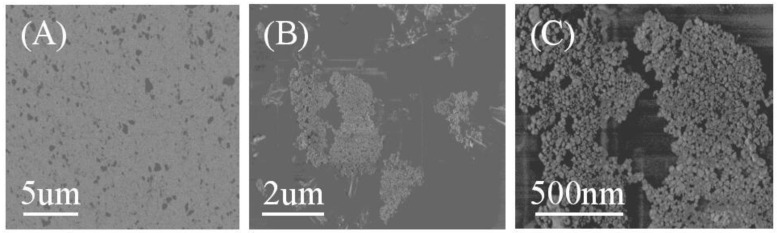
SEM images of bare GDE (**A**) and GDE after electrodeposition of Al(III) (**B**) and enlarge image of deposits (**C**). Films were electroplated at a potential of −1.80 V for 120 s from the extractant of 10 µg L^−1^ Al(III).

The stripping voltammograms of [C_8_mim][PF_6_], oxine in [C_8_mim][PF_6_], and the extractant of Al(III)**–**oxine at gold disk electrode were recorded and shown in Figure 1. Noticeably, there was no appreciable peak in the stripping voltammograms of [C_8_mim][PF_6_] (Figure 1A) and oxine in [C_8_mim][PF_6_] (Figure 1B); the anode peak at the potential of −1.1 V and −0.8 V emerged for the extractant of 1 ng L^−1^ Al(III) (C). These peaks should be oxidation peaks of Al deposits and are more positive than normal oxidation peak of Al, which could be due to alloy formation with the Au substrate during deposition. To further verify the hypothesis, the extractant of 10 µg L^−1^ Al(III) was transferred to the MVC and deposited at −1.8 V for 120 s. The SEM images and XRD analysis of the deposit are shown in Figure 2 and Supplemental Figure S2 (Supplementary Materials).

As shown in Figure 2A, the unmodified gold electrode surface is flat, and there are some small holes on the electrode surface. The hole increases the surface area of the electrode, which may increase the sensitivity of SW-ASV. Figure 2B shows the surface of a gold electrode prepared by electrodepositing the extractant of 10 µg L^−1^ Al(III). The surface is covered with a bulk grain particle, which should be aluminum deposits. The presence of Al deposits is more clearly from Figure 2C (enlarge image of deposits), and the diameters of the particles are between 20 nm and 50 nm. Energy dispersive spectroscopy (EDS) spectra are also presented in Figure S3 (Supplementary Materials) to validate the deposition of Al. EDS analysis displays the presence of 0.94% Al on the gold disk electrode surface after electrodeposition. As seen from Figure S2A (Supplementary Materials), a strong (111) diffraction peak of gold is obtained along with the other characteristic diffraction peaks (200), (220), and (311). Figure S2B (Supplementary Materials) shows the XRD patterns of a typical deposit obtained at a constant potential of −1.8 V for 120 s in [C_8_mim][PF_6_] after LPME of 10 µg L^−1^ aluminum on gold electrode. Compared with Figure S2A (Supplementary Materials), there is a shoulder peak at the right side of Au (111) diffraction peak, which might be the diffraction peak of the Au-Al alloy.

### 3.2. Factor of Al(III) Accumulation in SW-ASV

**Figure 3:**
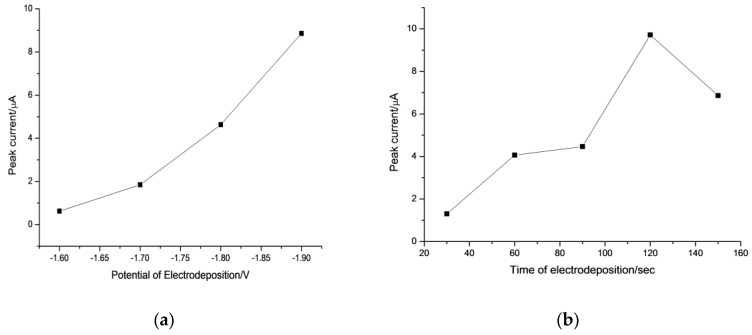
Effect of deposition potential (**a**) and deposition time (**b**) upon the SW-ASV response of 1 ng L^−1^ Al(III) after LPME on a GDE. In (a) deposition for 120 s; in (b) deposition at −1.8 V. LPME conditions: centrifugation rotor speed, 4500 rpm; extraction time, 10 min; pH of the sample, 6.5; sample volume, 30 mL; concentration of oxine, 0.67 mmol L^−1^; extraction IL, 150 µL. SW-ASV, quiet time: 10.0 s; frequency: 20.0 Hz; pulse amplitude: 25.0 mV; scan increment: 4.0 mV. All measurements were made in triplicate and the results were averaged.

The wider aim of this study was to expand the SW-ASV to detect pmol/L level of Al(III), which few previous reports have achieved. The low concentration of Al(III) forced us to find the best efficiency of LPME and SW-ASV; moreover, saturating electrode by higher concentration of Al(III) can be avoided. Concentration of 1 ng L^−1^ Al(III) was selected to optimize the experiment of LPME and SW-ASV. The effect of deposition potential on the peak current of 1 ng L^−1^ Al(III) after LPME was studied in the potential range from −1.5 to −2.0 V with 120 s of accumulation. Compared with the oxidation peak at −0.8 V, much less metal oxidized at −1.1 V, which was adopted in the experimental optimization and method evolution. As can be seen from Figure 3A, the oxidation peak of Al (at −1.1 V) were undetectable when deposition potentials were more positive than −1.60 V; this was because these potentials cannot initiate the reduction of Al(III). Peak currents of aluminum increased significantly after applying more negative potentials. Since no stirring was applied during SW-ASV, the rate of deposition is controlled primarily by diffusion and more negative electrolysis potential is in favor of preconcentration. However, the reproducibility of the signal of Al was poor at deposition potential of −1.9 V, which may result from the hydrogen evolution as the extractant contains a small amount of water. Based on these observations, deposition potential of −1.8 V was adopted in subsequent experiments. There are two advantages of using extractant to perform SW-ASV: first, hydrogen evolution is difficult in hydrophobic ILs, which allows us to carry out the experiments within a lower potential range and sensing highly active metal ions, such as K, Na, etc.; second, back-extraction is unnecessary since ILs can be adopted as electrolyte in SW-ASV, which simplify the operation. 

As depicted in Figure 3B, the effect of deposition time on the stripping performance for 1 ng L^−1^ of Al(III) was investigated in the range from 30 to 180 s. The stripping peak current of Al(III) increased with the increasing of deposition time up to 120 s, and a much wider peak was obtained for longer accumulation time. Hence, an accumulation time of 120 s was selected in the following measurements.

### 3.3. Optimization of IL-Based LPME

Several factors that influence the microextraction efficiency, such as concentration of the chelating agent and pH of the sample, were investigated. 

#### 3.3.1. Effect of Oxine Concentration

**Figure 4:**
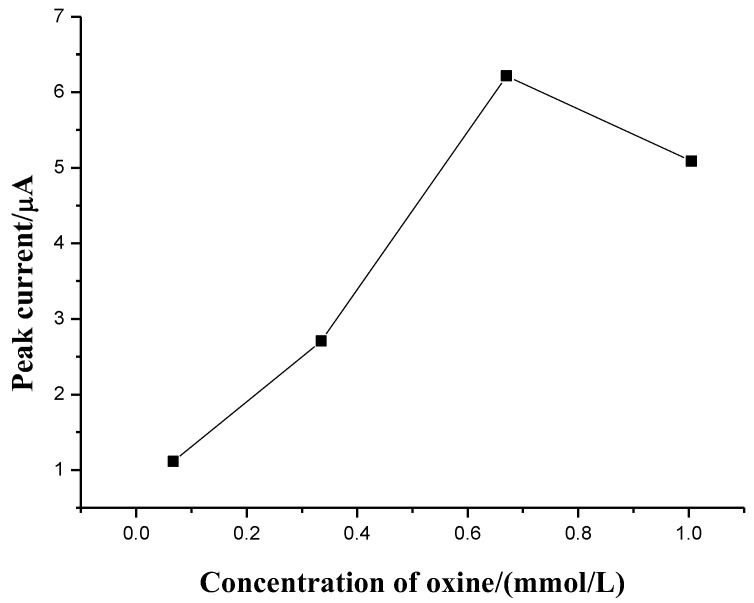
Effect of oxine concentration on the extraction efficiency of LPME. LPME conditions are as in Figure 3 except the concentration of oxine. Deposition potential: −1.8 V (vs. Ag/AgCl); deposition time: 120 s; quiet time: 10.0 s; frequency: 20.0 Hz; pulse amplitude: 25.0 mV; scan increment: 4.0 mV. All measurements were made in triplicate and the results were averaged.

Oxine is a ligand widely used to form chelates with transition and heavy metal cations. The chelates are hydrophobic and can extract into organic solvents/hydrophobic ILs during LPME. The effect of oxine concentration on the current of Al(III) was studied. Figure 4 shows the peak current of aluminum increased with the increasing concentration of oxine up to 0.67 mmol L^−1^. No further increase was observed for the concentrations above this value, which indicated that 0.67 mmol L^−1^ of oxine was sufficient for total complexation. The high amount of oxine offers satisfactory Al(III) recovery even in the presence of large excesses of other extractable species. Therefore, 0.67 mmol L^−1^ of oxine was selected to be an optimized concentration.

#### 3.3.2. Effect of pH

**Figure 5:**
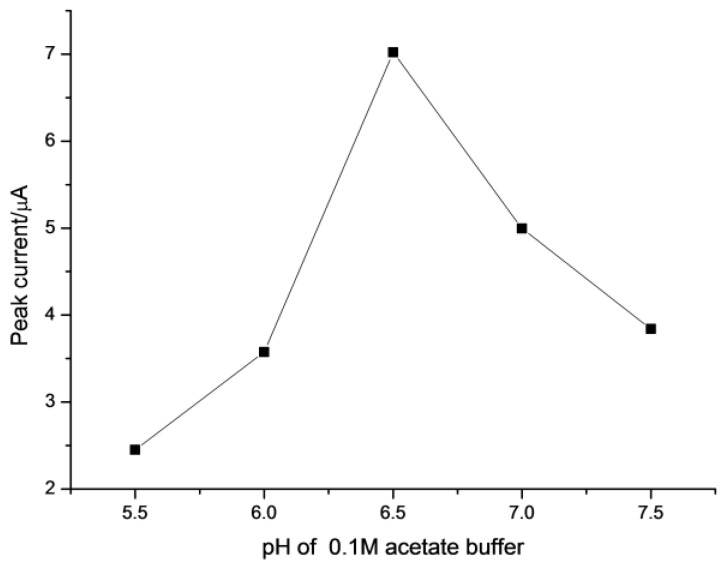
Effect of the pH on the LPME extraction efficiency of 1 ng L^−1^ Al(III). LPME conditions are as in Figure 3 except the pH of the solution. The parameters of SW-ASV are same as Figure 4.

The pH of the aqueous solution plays an important role in a metal–chelate formation and affects the subsequent LPME. In our experiment, the acetate acid buffer, which shows a fairly low level of alumimium contamination [61], was adopted as the supporting electrolytes. Figure 5 shows the effect of pH value of the solution on the signal intensity of Al(III). As can be seen from Figure 5, the peak of current increased as pH increased from 5.5 to 6.5 and started to decrease after pH 7.0. The reduced current in alkaline solution could be due to the formation of aluminum hydroxide, which decreases the amount of free Al^3+^ ions in sample. Hence, a pH of 6.5 was chosen for LPME.

### 3.4. Analytical Performance of the LPME–SW-ASV

**Figure 6:**
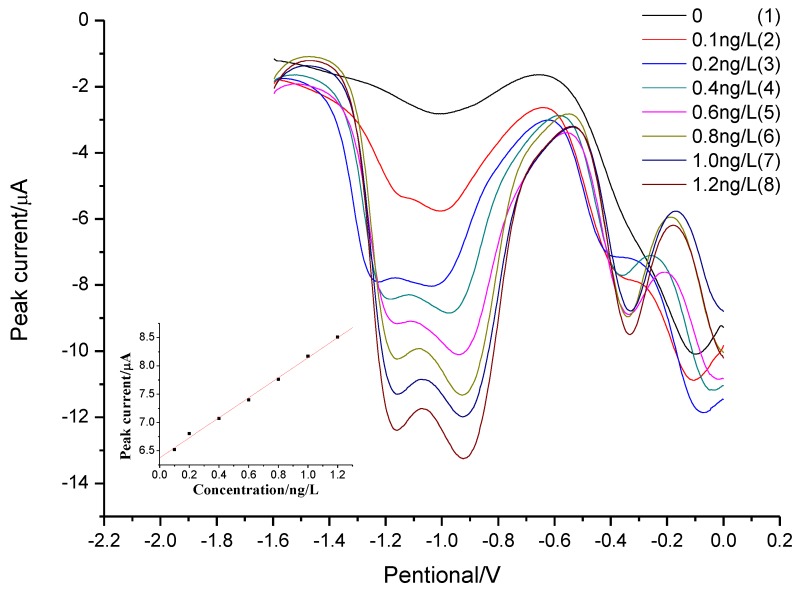
LPME-SW-ASV of Al(III) with different concentrations (from 0.1 to 1.2 ng L^−1^). Inset: Corresponding calibration plots. LPME conditions are same as Figure 3. The parameters of SW-ASV are same as Figure 4.

To evaluate the proposed method, the linearity, reproducibility, and limit of detection (LOD) were determined under optimal experimental conditions (Figure 6). Since the presence of ultratrace Al(III) in ultrapure water gave a blank signal, the peak current of individual standard sample was corrected manually by blank subtraction when the linear calibration cure was made. The calibration graph was linear in the range 0.1–1.2 ng L^−1^ for peak current of Al(III) at −1.1 V. The linear regression equation was *i_p_*_(Al(III))_(µA) = 6.5214 + 1.3977 [Al(III)](ng L^−1^) (*R^2^* = 0.9978, *n* = 3). The relative standard deviation (RSD) were 5.4% for ng L^−1^ Al(III) (*n* = 8). Since it is difficult to get a water without aluminum (c < 0.1 ng L^−1^) and no aluminum was found in ethanol, we use the mixture of oxine (dissolved in ethanol) and [C_8_mim][PF_6_] as the substitute for blank solutions. A horizontal baseline from the left base of the peak was adopted. The standard deviations of the blank at −1.1 V is 59 nA (N = 25), from which a detection limit of 0.023 ng L^−1^ (1 pmol L^−1^) (three times the standard deviations) was obtained. Ten times the standard deviation of the blank was used to evaluate the limits of quantification (LOQ). LOQ obtained was 0.076 ng L^−1^, which was close to the lower limit of linear range (0.1 ng L^−1^) of our method. These results proved we were not over-valuating the sensitivity of the method by using ethanol to simulate water without Al(III) and the LOD obtained is reasonable. To the best of our knowledge, it provided the lowest DL for aluminum detection (Table 1).

The effect of foreign ions on the recovery of Al(III) was studied by adding the investigated ions to a solution containing 0.1 ng L^−1^ of Al(III), and the recommended procedure was followed. The tolerance limits—defined as the highest amount of foreign ions that changed the peak current of Al(III) by five percent—were presented in Table 2. The interference experiment will stop when the interferential concentration is 5000-fold higher than that of Al(III). As can be seen from the Table 2, the following ions, such as K^+^, Na^+^, Zn^2+^, Pb^2+^, Cd^2+^ and Cr^3+^, had little effect on the determination of aluminum(III). Compared with the previous reports using AdSV detection, the interference from the common metal ions remarkably decreased [15,22]. This can be attributed to the following factors: the metal ions, which cannot form a metal ion–oxine complex and have the poor solubility in [C_8_mim][PF6], would be eliminated during LPME; moreover, the oxidation peak of the most metals, such as Zn, Pb, and Cd, is more positive than that of Al, and can be easily discriminated from Al during SW-ASV analysis.

### 3.5. Analytical Application to Commercial Distilled Water

**Figure 7:**
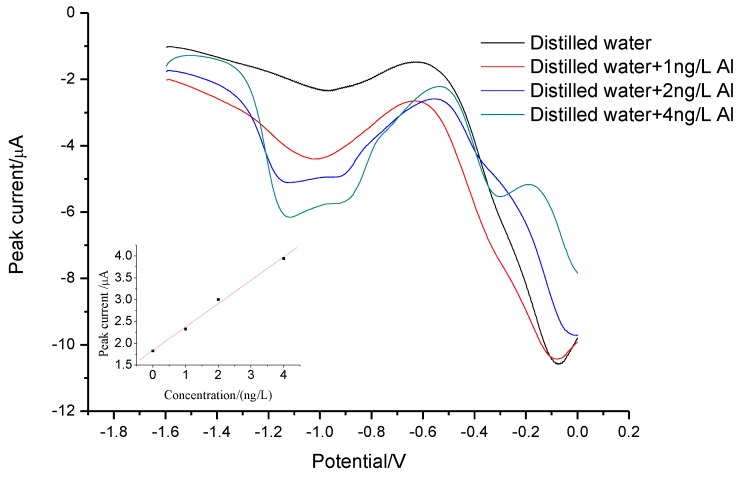
LPME**–**SW-ASV of Al(III) in commercial distilled water sample with standard addition method. The inset shows the corresponding standard plot. LPME conditions are same as Figure 3. The parameters of SW-ASV are same as Figure 4.

The method was applied to detecting Al(III) in a commercial distilled water. Since the amount of Al(III) in sample is beyond the upper limit of the method, 0.3 mL of a commercial distilled water was used instead of 30 mL. As depicted in Figure 7, the regression equation was *i_p_*(Al(III))(µA) = 1.833 + 0.536[Al(III)](ng L^−1^) (*R^2^* = 0.9948). The concentration of Al(III), evaluating by extrapolation, was 3.42 ± 0.63 ng L^−1^. The recoveries of aluminum were between 91.0% and 109% for spiked samples. The ICP-MS yielded a concentration of Al(III) equal to 4.2 ± 1.2 ng L^−1^. The present method was in agreement with those determined by ICP-MS, which proves the accuracy of the proposed methodology.

## 4. Conclusions

Liquid phase microextraction coupled with anodic stripping voltammetry used to determine ultra-trace aluminum in aqueous samples was reported for the first time. It has the following important merits: (a) Our method provides better selectivity for Al(III) due to the selective extraction of oxine-complexes in water samples during LPME and the chemical resolution of SW-ASV; (b) The LOD attains as low as 1 pmol L^−1^, which reaches the lowest report for Al(III) detection using electroanalytical techniques; moreover, the lower limit of linear range (0.1 ng L^−1^) in our experiment was even lower than the lowest LOD for Al(III) reported using electrochemical detection; (c) Reusable ionic liquid and non-mercury electrode was adopted in our experiment, and the method is environment friendly; (d) The method can be applied to analyze other metal ions with high electronegativity by choosing appropriate chelating agent, which provides an attractive alternative to cathodic adsorptive stripping voltammetry.

## Figures and Tables

**Table 1:** Comparison of the proposed method with other electrochemical techniques for the determination of Al(III).

Technique	Modification/Chelator	LOD (nM)	Reference	Technique	Modification/Chelator	LOD (nM)	Reference
Mercury Drop Electrodes				Glass Carbon Electrode			
AdSV	SVRS	76	[16]	DPV	Alizarin (graphene on E)	90	[35]
AdSV	cupferron	50	[30]	AdSV	Cupferron (Bismuth film GCE)	18	[32]
AdSV	oxine	85	[25]	DPV	alizarin red S on E	80	[36]
AdSV	dithiooxamide	0.4	[27]	Amperometry	DASA	370	[24]
SW-AdSV	oxine	0.7	[26]	DPV	catechol	1.4	[12]
AdSV	SVRS	67	[17]	DPV	L-dopa	890	[38]
AdSV	pyrogallol red	37	[28]	DPV	dopamine	140	[39]
AdSV	DASA	30	[20]	DPV	L-dopa	76	[40]
LSV	norepinephrine	1800	[37]	AdSV	TMAC	0.05	[10]
AdSV	SVRS	52	[18]	Carbon electrode			
AdSV	SVRS	3.8	[15]	ASV	Zinc	296	[8]
AdCP	PCV	8	[14]	Potentiometry	AlMCM-41 on E	460	[11]
AdSV	morine	4.07	[29]	Gold nanoparticles modified carbon electrode			
AdSV	Alizarin S	25	[33]	Amperometry	AChE on E	2100	[42]
DP-AdSV	DASA	7.4	[21]	Amperometry	α-chymotrypsin on E	3300	[43]
AdSV	DASA	1.8	[22]	Pyrolytic graphite electrode			
Mercury film modified glass carbon electrode				DPV	PCV on E	5	[13]
SWV	Alizarin R	10	[34]	Voltammetry	SVRS	370	[19]
AdSV	DASA	1000	[23]	Gold electrode			
LSV	cupferron	18	[31]	EIS	SHQ	0.008	[45]
Bismuth film on Pt electrode				SW-ASV	Oxine	0.001	This work
AdSV	EBBR	0.56	[41]				

AdCP: Adsorption Chronopotentiometry, DPV: differential pulse voltammetry, EIS: electrochemical impedance spectroscopy, LSV: linear scan voltammetry, SWV: square wave voltammetry; AChE: Acetylcholinesterase, DASA: 1,2-dihydroxyanthraquinone-3-sulfonic acid, EBBR: Eriochrome blue black R; PCV: pyrocatechol violet, SVRS: solochrome violet RS, TMAC: tetramethylammonium chloride.

**Table 2:** Tolerance limits of foreign ions in the determination of 0.1 ng L^−1^ Al^3+^. All measurements were made in triplicate and the results averaged.

Element	Tolerance Limit (ng L^−1^)	Interferent: Al Ratio
K^+^, Na^+^	500	5000
Cd^2+^, Pb^2+^, Cr^3+^, Zn^2+^	100	1000
Ca^2+^, Cu^2+^, Sn^2+^	10	100

## Supplementary Materials

The following are available online at http://www.mdpi.com/1424-8220/18/5/1503/s1, Figure S1: Image of microliter voltammetric cell from top view (**A**) and side view (**B**). Figure S2: XRD patterns of bare gold disk electrode (lower curve) and the deposit obtained potentiostatically at −1.8 V for 120 s after IL-based LPME of 10 µg L^−1^ Al(III) on the gold substrate (upper curve). Figure S3: Energy-dispersive X-ray spectroscopy (EDX) analysis of Al deposition on GDE.
