# Supplementary material for: Quantitative Determining of Ultra-Trace Aluminum Ion in Environmental Samples by Liquid Phase Microextraction Assisted Anodic Stripping Voltammetry
Source: Sensors (Basel). 2018 May 10;18(5):1503. doi: 10.3390/s18051503 (PMC5981444; doi:10.3390/s18051503)
Supplement: Supplementary file 1 [file sensors-18-01503-s001.pdf]

Supplementary Materials: Quantitative Determining of Ultra-Trace Aluminum Ion in Environmental Samples by Liquid Phase Microextraction Assisted Anodic Stripping Voltammetry

Liuyang Zhang [1,†], Jinju Luo [1,†], Xinyu Shen [2], Chunya Li [1], Xian Wang [1], Bei Nie [1] and Huaifang Fang [1,*]

1 Key Laboratory of Analytical Chemistry of the State Ethnic Affairs Commission, College of Chemistry and Materials Science, South-Central University for Nationalities, Wuhan 430074, China; zlyzly1239@163.com (L.Z.); JinjuLuo@163.com (J.L.); lichychem@163.com (C.L.); xwang27@hotmail.com (X.W.); bnie@cigit.ac.cn (B.N.)
2 Key Laboratory of Analytical Chemistry for Biology and Medicine, Wuhan University, Ministry of Education, Wuhan 430072, China; shenxy@whu.edu.cn
* Correspondence: hffang@mail.scuec.edu.cn; Tel.: +86-135-1729-5825
† These authors contributed equally to this work.

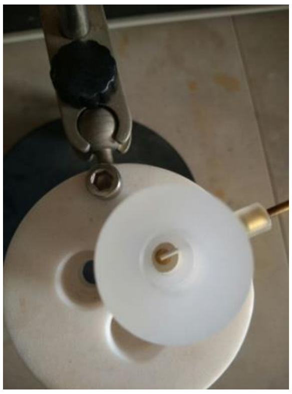

(A)

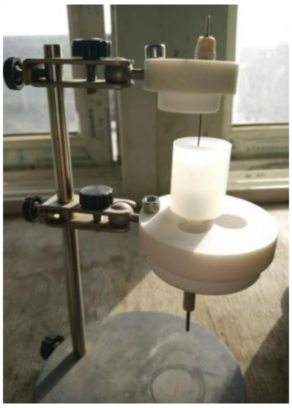

(B)

Figure S1. Image of microliter voltammetric cell from top view (**A**) and side view (**B**).

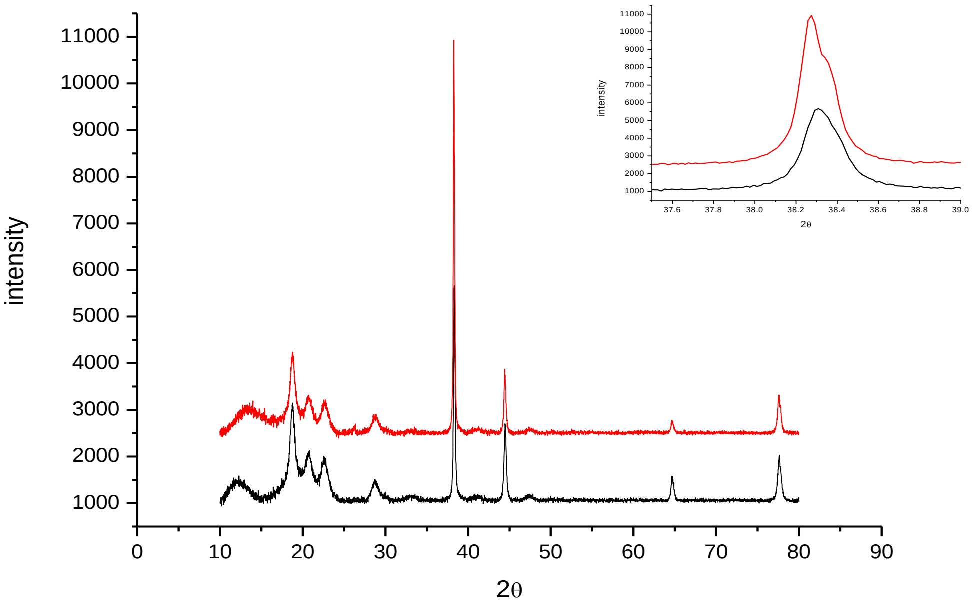

Figure S2. XRD patterns of bare gold disk electrode (lower curve) and the deposit obtained potentiostatically at −1.8 V for 120 s after IL-based CME of 10 μg L^{-1} aluminium on the gold substrate (upper curve). Inset: enlarged image of Au(1 1 1) peak of XRD patterns.

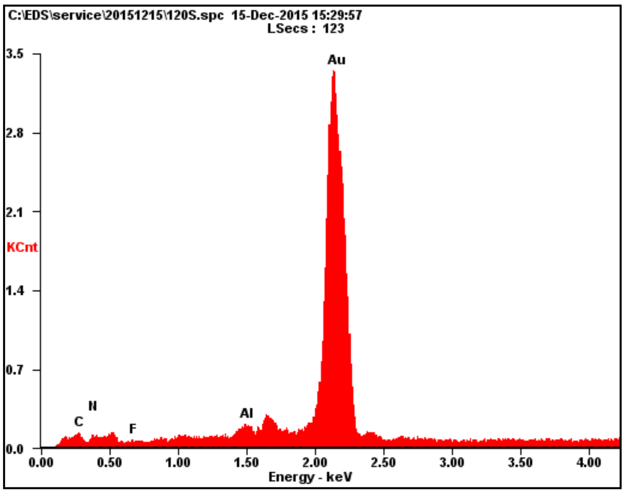

Element	*Wt %*	*At %*
C K	10.59	45.45
N K	08.45	31.09
F K	00.29	00.80
AlK	00.94	01.80
AuL	79.72	20.86

Figure S3. Energy-dispersive X-ray spectroscopy (EDX) analysis of Al deposition on GDE.
